# The collapse of the food matrix: how ultra-processed foods impact satiety and metabolism by altering physical structure beyond nutrient composition

**DOI:** 10.3389/fnut.2026.1737280

**Published:** 2026-05-05

**Authors:** Xinna Wang, Hang Chen, Yan Xu, Qiaoli Xu, Hongtao Cui, Fang Liu

**Affiliations:** 1 Department of Encephalopathy, Affiliated Hospital of Changchun University of Chinese Medicine, Changchun, China; 2 Department of Pediatrics, The First Affiliated Hospital, Henan University of Chinese Medicine, Zhengzhou, Henan, China; 3 School of Pediatrics, Henan University of Chinese Medicine, Zhengzhou, Henan, China; 4 Department of Pediatrics, Chongqing Traditional Chinese Medicine Hospital, Chongqing, China; 5 The First Affiliated Hospital of Chongqing University of Chinese Medicine, Chongqing, China; 6 Department of Endocrinology and Metabolism, Chongqing Traditional Chinese Medicine Hospital, Chongqing, China

**Keywords:** food matrix, metabolism, physical structure, satiety, ultra-processed foods

## Abstract

The global consumption of ultra-processed foods (UPFs) is strongly associated with the prevalence of non-communicable diseases (NCDs). This link has traditionally been attributed to their poor nutritional profiles. However, evidence shows that even when nutrient-matched, UPFs promote excess energy intake and weight gain, suggesting a pathogenic mechanism beyond their chemical composition. This review proposes a central conceptual framework: the core threat of UPFs to health may originate profoundly from the industrial collapse of their physical “food matrix.” While evidence-informed, this framework remains a conceptual proposition requiring further causal validation. We hypothesize that this structural disintegration triggers a proposed top-down cascade of dysregulation. In the oral phase, a soft matrix accelerates eating rates by reducing chewing requirements, thereby weakening early satiety signals. In the gastrointestinal tract, the excessively rapid absorption of nutrients suppresses the secretion of distal gut satiety hormones, such as glucagon-like peptide-1 (GLP-1) and peptide YY (PYY). This supraphysiological nutrient flux imposes a significant challenge on core metabolic organs, driving insulin resistance and hepatic *de novo* lipogenesis. Ultimately, the impoverished matrix leads to gut microbiota imbalance, compromised intestinal barrier function, and low-grade systemic chronic inflammation. In conclusion, the integrity of the food matrix is an indispensable dimension for evaluating the health value of food. This paper calls for a fundamental shift in perspective within nutritional science and public health policy: from focusing solely on “what is in our food” to equally considering “what has been done to our food.”

## 1 Introduction

Over the past half-century, human societies have undergone a profound “Nutrition Transition,” characterized by a rapid shift from traditional, plant-based, minimally processed diets to “Westernized” diets high in energy density, animal-derived products, and industrial processing (1). In the later stages of this transition, the rise and proliferation of ultra-processed foods (UPFs) have become the most prominent phenomenon and a primary driver (1, 2). These ready-to-eat or ready-to-heat industrial formulations are replacing traditional meals at an unprecedented scale. In parallel, the global burden of non-communicable diseases (NCDs) has shown an exponential increase. A large and highly consistent body of epidemiological research has conclusively established a strong, dose-dependent positive correlation between higher UPF consumption and an increased risk of multiple adverse health outcomes. For instance, the latest meta-analyses show that compared to the lowest consumption group, the highest consumers of UPFs have a 15% increased risk of all-cause mortality (HR = 1.15, 95% CI 1.09–1.22) (3) and an 11% increased risk of cardiovascular disease (HR = 1.11, 95% CI 1.06–1.16) (4). Furthermore, each 10% increase in UPF intake is associated with a 10% rise in the risk of type 2 diabetes (RR = 1.10, 95% CI 1.08–1.12) (5). Experimental evidence demonstrates that the soft texture, high energy density and hyperpalatable nutrient combinations of UPF facilitate excessive energy intakes by affecting ingestive behaviors, satiety signaling and food reward systems (6). To stem the global rise in obesity, multipronged policy efforts are needed to reduce UPF consumption and create health-promoting food systems. Moreover, ultra processed foods cause conformational changes in proteins, thereby affecting the body’s sensitivity to allergens (7). In response to this severe public health challenge, the mainstream explanatory framework in academia and policy has long focused on their adverse nutritional composition: high levels of added sugars, unhealthy fats, and sodium, coupled with a deficiency in dietary fiber and micronutrients (8).

Although this nutrient-based “reductionist” explanation provides the theoretical foundation for current dietary guidelines, its explanatory power is facing increasing challenges. A growing body of evidence suggests that nutrition labels alone cannot fully account for the observed health risks. Among these, a landmark randomized controlled trial conducted by Kevin Hall’s team posed a decisive challenge to this traditional paradigm (9). In this study, participants were randomly assigned to either an ultra-processed or an unprocessed diet group, with both diets meticulously matched for energy density, macronutrients, sugar, sodium, and dietary fiber. The results clearly demonstrated that participants on the ultra-processed diet consumed, on average, approximately 500 kcal more per day *ad libitum*, accompanied by significant weight gain. While this landmark trial provides robust causal evidence linking UPF consumption to excess energy intake and short-term weight changes, it is important to clarify that the downstream mechanisms proposed in this review—such as specific endocrine responses, microbial remodeling, and inflammatory cascades—were not directly measured in Hall’s study. Rather, these subsequent pathways are inferred from a synthesis of separate mechanistic and *in vivo* studies. This “anomalous” finding, inexplicable by differences in nutrient composition, provides compelling evidence for a deeper pathogenic factor that transcends the chemical makeup of food. This factor is the food’s physical structure, known as the “food matrix” (10). The food matrix is defined as the physical organization of nutrient and non-nutrient components within a food and their complex interactions. It determines the bioaccessibility and absorption kinetics of nutrients, thereby holistically modulating the body’s physiological responses—a phenomenon termed the “Food Matrix Effect” (11).

Natural foods possess an intact and complex matrix. In contrast, the fundamental commercial purpose of ultra-processing techniques—such as extrusion, puffing, pre-gelatinization, and refining—is to deliberately and systematically deconstruct this natural barrier to create industrial products with extended shelf life, hyper-palatability, and high convenience. This process is essentially a form of “industrial pre-digestion”—defined here as the external application of mechanical and chemical forces to break down food structures, a process the human gastrointestinal tract is evolutionarily designed to perform internally. Therefore, this review proposes and aims to systematically indicates a core argument: the fundamental health threat of UPFs stems not merely from the simple addition or subtraction of nutrients, but from the “structural collapse” of the physical matrix driven by industrialization. It is this structural disintegration that allows nutrients to bypass the sophisticated digestive and absorptive regulatory mechanisms evolved over millions of years to handle natural foods, thereby systematically disrupting inherent satiety signaling pathways and core metabolic homeostasis.

**FIGURE 1:**
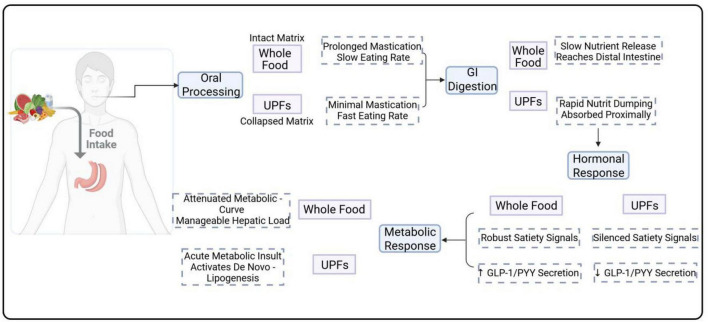
Comparative diagram of the impact of food matrix integrity on digestion and metabolism.

Consequently, the primary purpose of this review extends beyond merely summarizing existing evidence; it aims to establish and advocate for a new food health assessment framework centered on “food matrix integrity,” thereby promoting a necessary re-evaluation in nutritional science. This paper will systematically integrate evidence from various fields to demonstrate how the physical form of UPFs determines their profound impact on satiety, metabolism, and inflammation through a series of cascading reactions. The theoretical contributions and practical implications of this new framework are multi-faceted: (1) For scientific research, it calls for methodological innovation, demanding that physical structure be given equal importance to chemical composition when evaluating the health effects of food, and that experiments be designed to disentangle these two effects. (2) For public health policy, it raises fundamental questions about the adequacy of current nutrient-based food labeling systems (e.g., “Health Star Ratings”) and provides a strong theoretical basis for incorporating “degree of processing” as a core consideration in national dietary guidelines. (3) For clinical practice and public education, it offers a simpler, more intuitive, and more actionable principle for healthy eating—prioritizing “whole foods” with the lowest degree of processing. (4) For the food industry, it points toward a future direction for innovation: the development of “gentle” processing technologies hat better preserve the natural food matrix. By shifting the academic and public focus from “what is in our food” to “what has been done to our food,” this review provides a solid theoretical foundation and a clear practical roadmap for more effectively addressing the global challenge of NCDs, see as Figure 1.

## 2 Oral processing and eating behavior: how the first line of satiety defense is compromised

The initial physiological impact of the food matrix occurs in the oral cavity, a critical stage that determines energy intake efficiency and the strength of early satiety signals. An intact physical structure mandates a prolonged “oral residence time,” providing valuable time for the activation of complex neuroendocrine satiety signaling networks and forming the first physiological defense against excessive energy intake. The harm of UPFs lies in their systematic dismantling of this structure, which simultaneously compromises this defense system at biomechanical, neurophysiological, and behavioral levels.

The matrix of whole foods, whether the toughness of plant cell walls or the density of meat muscle fibers, requires thorough mastication to complete its “food breakdown dynamics” in the mouth—a gradual comminution and mixing with saliva to form a swallowable bolus (11). This obligatory mechanical processing directly governs two interrelated mechanisms of early satiety regulation. First, at the behavioral level, it increases the number of chews per unit of food, directly and significantly slowing the eating rate (12). A slower eating rate is a critical time window for satiety generation, allowing sufficient time for the subsequent release of gastrointestinal satiety hormones (e.g., cholecystokinin, CCK) and the transmission of vagal afferent signals to the brain, enabling the body to promptly perceive energy intake and generate a feeling of fullness (13). Second, at the neurophysiological level, prolonged chewing and slow food breakdown greatly increase the food’s “oro-sensory exposure.” This refers not only to taste and smell but, more critically, to the sustained and varied mechanical stimulation of the oral mucosa, teeth, and tongue, known as “mouthfeel” (14). This rich and prolonged sensory input, transmitted via pathways like the trigeminal nerve, strongly activates the central nervous system and is a core prerequisite for effectively triggering the “Cephalic Phase Response” (15). This response, an important “feed-forward” regulatory mechanism, pre-emptively signals the digestive tract to secrete digestive juices, increase blood flow, and initiate early-phase insulin release, thereby preparing the entire metabolic system for the impending nutrient load.

In stark contrast, a key design philosophy of UPFs is to maximize “ease of eating.” Their soft, crispy, pre-gelatinized, or liquid matrices are direct products of industrial deconstruction, with the primary goal of minimizing the need for chewing and thus shortening oral processing time. This disrupted matrix undermines both of the aforementioned mechanisms. On one hand, it leads to a sharp acceleration in eating rate. Studies confirm that even consciously controlling chewing frequency significantly alters oral processing and texture perception (16), while the soft matrix of UPFs unconsciously and obligatorily reduces chewing demand, leading to an uncontrolled eating rate. This is perfectly consistent with the observations of Hall’s team: participants consuming a UPF diet had a significantly higher eating rate (g/min) than those on an unprocessed diet, which was highly correlated with the eventual excess intake of approximately 500 kcal per day (9). On the other hand, the brief oral residence time and the impoverished, monotonous mechanical sensory stimulation severely attenuate the intensity and duration of the cephalic phase response. The ultimate result is that a large quantity of calories is rapidly ingested before the body’s satiety “defense system” is fully activated. This constitutes the first “insult” to energy homeostasis, opening the door for subsequent energy imbalance and metabolic dysregulation.

## 3 Gastrointestinal dynamics and hormonal response: the silenced intestinal “brake”

After leaving the oral cavity, the structural differences in the food matrix trigger more profound consequences for digestive dynamics and endocrine responses within the gastrointestinal tract. The core principle is that an intact matrix acts as a sophisticated “nutrient slow-release system,” ensuring through multiple mechanisms that nutrients are released slowly and continuously, allowing them to interact with “nutrient sensors” in the distal intestine. Conversely, a collapsed matrix acts like a “nutrient flood,” overwhelming and silencing the gut’s inherent hormonal feedback systems.

The critical link between oral processing and intestinal response is the rate of gastric emptying. The physical form of food, particularly its viscosity, particle size, and solid-to-liquid ratio, is a primary regulator of gastric emptying. Whole foods with an intact matrix and higher viscosity significantly delay the passage of gastric contents into the duodenum, constituting a key “rate-limiting step” for nutrient absorption (17). For example, foods rich in soluble fiber (like oats) can form a high-viscosity gel in the stomach, slowing emptying by activating gastric wall mechanoreceptors. This delaying effect, combined with the physical barrier of the matrix itself (e.g., the encapsulation of starch by intact plant cell walls), ensures that a substantial portion of incompletely digested nutrients can “survive” and successfully “travel” to the distal small intestine—the ileum.

This is precisely the physiological basis for activating the “ileal brake” mechanism (18). The ileum is one of the body’s most powerful satiety regulation centers, its walls densely populated with specialized endocrine L-cells for “nutrient sensing.” These cells act like sentinels, monitoring the chemical environment of the intestinal lumen. When undigested carbohydrates and fats arrive, they constitute the strongest physiological stimulus, activating molecular sensors like G-protein coupled receptors (e.g., GPR40/41/43/120) and triggering the L-cells to secrete large amounts of two key anorexigenic hormones: glucagon-like peptide-1 (GLP-1) and peptide YY (PYY) (19). Once in circulation, these hormones act directly on the appetite centers of the hypothalamus to produce a strong sense of satiety. They also form a powerful negative feedback loop by slowing gastric emptying and intestinal motility, effectively terminating eating behavior.

The danger of UPFs lies in their systematic disruption of this precise mechanism. Their collapsed, low-viscosity matrix properties lead to a double blow of “rapid gastric emptying” and “rapid proximal small intestine absorption.” Grinding, homogenization, and extrusion leave nutrients fully “exposed,” making them easily and completely digested and absorbed in the upper duodenum and jejunum. This “proximal shift of absorption,” caused by matrix collapse, is a critical pathogenic step of UPFs (10). Because virtually no nutrients reach the “sensing region” of the ileum, the L-cells receive no stimulation, leading to a severe suppression of postprandial GLP-1 and PYY secretion. This silencing of the “ileal brake” not only directly weakens intra-meal satiety, thereby permitting excess energy intake, but more importantly, the lack of GLP-1 secretion impairs its crucial function as an “incretin”—promoting insulin secretion and suppressing glucagon release in response to glucose. This not only weakens the body’s ability to dispose of glucose but also sets the stage for the acute metabolic insult driven by rapid absorption, which will be discussed in the next section.

Only studies that actively manipulate or compare food physical structure while controlling nutrients provide causal evidence for the matrix hypothesis (see Table 1). Future research should integrate objective measures of food structure (e.g., texture analysis *in vitro* digestion kinetics into UPF assessments.

**TABLE 1:** Evidence linking food matrix disruption to health outcomes.

Study	Key findings	Critical parameters of study/experimental design	Limitations
Li et al. (20)	Each 10% increase in UPF energy intake associated with higher HOMA-IR and fasting insulin in adolescents, even after adjusting for total energy, sugar, fat, and fiber intake.	• Longitudinal cohort with follow-up (*n* = 85 completed) • UPF quantified via NOVA classification from 24-h recalls • Insulin resistance measured via HOMA-IR • Adjusted for multiple dietary and lifestyle covariates	• Small final sample (*n* = 85) • Single time-point biomarker measurement • NOVA does not capture food texture or matrix integrity
Åberg et al. (21)	Consuming minimally processed, intact whole grains significantly improved daily glycemic control and reduced postprandial blood glucose spikes compared to nutrient-matched finely milled whole grains.	• Single-arm prospective pilot study with embedded non-randomized IF vs. control subgroup comparison. • 42 adults with type 2 diabetes (T2D) and BMI ≥ 25 kg/m^2^; 34 completed the 6-month program. Personalized Mediterranean-style nutrition counseling + supervised aerobic/resistance training (3 × /week), supported by digital monitoring (heart rate sensors, app). • Primary—feasibility; Secondary—HbA1c, anthropometrics, VO2 max, blood lipids; Exploratory—dietary intake (3-day records), body composition.	• Small sample size and lack of randomization limit causal inference, especially for IF subgroup analysis. • No passive control group; all participants received intensive intervention. • Short duration (6 months) precludes assessment of long-term sustainability.
Sommersten et al. (22)	Diets with high “carbohydrate cellularity” (intact plant cells) improved postprandial glycemia and satiety vs. nutrient-matched low-cellularity diets, despite identical macronutrient composition.	• Randomized crossover trial • Controlled for sex and couple status in randomization • Used baseline-adjusted constrained linear mixed-effects models (cLMMs) • Diets matched for energy, protein, fat, fiber, but differed in physical structure (whole vs. pureed/milled)	• High dropout rate → potential selection bias • Short intervention period (weeks) • Limited generalizability beyond metabolic health markers
Grundy et al. (23)	The physical intactness of the cell wall matrix directly dictates lipid bioaccessibility. Meals with less disrupted matrix (larger particles) significantly attenuated postprandial lipemia.	• Prospective cohort (*n* = 7,000 +) • Dietary assessment via repeated FFQ • Cognitive function measured annually with validated tests • Adjusted for education, physical activity, comorbidities	• Observational → residual confounding likely • No data on food physical properties or chewing behavior
Zaretsky et al. (24)	UPF diet impaired bone microarchitecture (↓ trabecular thickness, ↑ porosity) and mechanical strength in rats, even when calories and calcium/phosphorus were matched to control. Effects linked to growth plate disruption and systemic inflammation.	• Controlled animal experiment (rats) • UPF group vs. control + Ca/P-matched group • μCT for bone structure, biomechanical testing, serum cytokines • Diets matched for Ca, P, energy	• Animal model → limited human translatability • UPF formulation may not reflect real-world products • Short duration (weeks)
Moretti et al. (25)	• Iron bioavailability varied dramatically by food matrix: milk strongly inhibited Fe absorption (∼2% RBV), while meat/bread enhanced it (up to 62% RBV). Vitamin C partially overcame inhibition.	• Crossover design in iron-deficient women (*n* = 26) • Used isotopically labeled [57Fe]-MDFP and [58Fe]-ferrous sulfate • Measured erythrocyte incorporation after 14 days • Compared same iron compound in different matrices (milk cereal vs. rice meal)	• Small sample size • Focused only on iron; not generalizable to other nutrients or chronic outcomes • Did not test ultra-processed forms directly

## 4 Metabolic impact: from nutrient flood to organelle stress

The harm of food matrix collapse is not just about “how much is eaten,” but more critically, “how fast it is absorbed.” This “Nutrient Flood”—defined in this review as the abnormally rapid, synchronous, and massive influx of macronutrients into the portal and systemic circulation due to the loss of natural absorptive barriers—imposes an unprecedented “Nutrient Overload” on the core metabolic hubs within cells, particularly the mitochondria and endoplasmic reticulum of the liver. This triggers a series of organelle stress responses, which are the fundamental drivers of the core pathophysiological processes of NCDs.

For carbohydrates, the integrity of the matrix directly determines their glycemic index. Starch physically encapsulated by the matrix is hydrolyzed slowly, resulting in a gentle postprandial blood glucose curve. In contrast, the “free” sugars and refined starches in UPFs cause a sharp spike in blood glucose followed by hyperinsulinemia (26). This high-intensity, pulsatile insulin stimulation, driven by the ultra-fast absorption rate, is a key initiating factor in the development of insulin resistance in peripheral tissues (such as muscle and fat). At the molecular level, sustained hyperinsulinemia induces the degradation of the insulin receptor (IR) and its substrate (IRS-1) through negative feedback mechanisms and activates protein tyrosine phosphatases (like PTP1B), thereby systematically blunting the insulin signaling pathway.

For lipids, the natural food matrix (e.g., milk fat globule membrane, plant cell walls) also plays a crucial role as a “digestive barrier,” effectively slowing the access and action of lipases and enabling slow fat absorption. In contrast, the industrially homogenized and pre-emulsified fats in UPFs bypass this physiological barrier, leading to severe “postprandial lipemia.” This supraphysiological flux of fatty acids, along with excess substrates from carbohydrates, is preferentially converted into triglycerides in the liver. This process strongly activates the “*De Novo* Lipogenesis” (DNL) pathway. The core mechanism involves excess glucose and insulin synergistically upregulating the activity of key transcription factors ChREBP and SREBP-1c, which in turn drives the expression of rate-limiting enzymes like acetyl-CoA carboxylase (ACC) and fatty acid synthase (FAS), ultimately leading to massive fat accumulation in the liver. This constitutes the core biochemical mechanism linking UPFs to non-alcoholic fatty liver disease (NAFLD) and hypertriglyceridemia (27).

Ultimately, these macroscopic “nutrient floods” converge at the microscopic organelle level, inflicting a devastating blow on mitochondria and the endoplasmic reticulum. When an overload of nutrient substrates (glucose, fatty acids) simultaneously floods the mitochondria, its tricarboxylic acid cycle and electron transport chain (ETC) become overwhelmed. This leads to a massive “leakage” of electrons during transport, which react with oxygen to form superoxide and other reactive oxygen species (ROS), causing severe oxidative stress. This mitochondria-derived oxidative stress not only directly damages mitochondrial DNA and proteins but, more importantly, it activates stress kinase pathways such as JNK and IKKβ. Activated JNK can directly phosphorylate inhibitory serine sites on the insulin receptor substrate (IRS-1), thereby blocking normal insulin signal transduction. This perfectly explains at a molecular level how “fast nutrients” directly cause insulin resistance. Concurrently, excessive DNL and protein misfolding can also induce endoplasmic reticulum stress (ER Stress), further exacerbating inflammation and insulin resistance. Thus, by creating a series of “metabolic insults” against organelles, the collapse of the food matrix transforms a meal from an orderly “nutrient supply” into an attack on the entire body’s cells, causing oxidative damage and functional dysregulation.

## 5 Gut microbiome remodeling: a “dual hit” of matrix deprivation and chemical assault

The food matrix is not only a source of nutrition for the host but also the “habitat” and “food” for the trillions of microorganisms in the gut. UPFs inflict a devastating impact on this complex ecosystem through a “dual-hit” model: on one hand, they “deprive” it of essential natural substrates, and on the other, they “inflict” a direct chemical assault. Together, these actions dismantle the gut barrier and ignite the fuse of systemic inflammation.

The first hit is “matrix deprivation.” A healthy gut microbiota depends on a diverse array of indigestible “Microbiota-Accessible Carbohydrates” (MACs) (28). In whole foods, these MACs exist as complex, heterogeneous polysaccharide networks (e.g., pectin, hemicellulose, resistant starch) within intact plant cell walls, providing rich ecological niches and fermentation substrates for various functional microbes. Ultra-processing completely destroys these structures. Even when single, refined fibers (like inulin) are “added back,” they cannot replicate the physical structure and chemical diversity of the original matrix. This extreme simplification and impoverishment of substrates leads directly to a “famine” for key beneficial bacteria, such as butyrate producers, and a sharp decline in the production of short-chain fatty acids (SCFAs). Butyrate is not only the preferred energy source for colonocytes but is also a critical signaling molecule that maintains the integrity of the gut barrier’s tight junctions (by upregulating proteins like Occludin and Zonulin-1) and suppresses inflammation by activating the GPR109A receptor (29). Therefore, by depriving beneficial bacteria of their survival base, the collapse of the food matrix indirectly but powerfully weakens the physical and immunological integrity of the gut barrier.

The second hit is a “chemical assault,” primarily from the non-nutritive food additives prevalent in UPFs. These substances are not inert; they have a direct destructive effect on the gut microbiota and barrier function. Emulsifiers are a classic example. Groundbreaking research has shown that common food emulsifiers, such as carboxymethylcellulose and polysorbate-80, act like surfactants or “detergents.” They can directly erode and disrupt the mucus layer composed of MUC2 mucin, weakening its physical barrier function and allowing gut bacteria to come into closer contact with intestinal epithelial cells. This abnormal contact triggers pro-inflammatory signaling pathways, leads to gut dysbiosis, and has been shown to directly induce chronic colitis and metabolic syndrome in animal models (30). Furthermore, some non-nutritive sweeteners, such as sucralose, have been shown to selectively inhibit the growth of certain beneficial bacteria, thereby significantly altering the composition and function of the gut microbiota and even inducing glucose intolerance in some individuals by affecting the microbiome (31).

In summary, UPFs create an intestinal “ecological catastrophe” through the dual mechanisms of “depriving beneficial substrates” and “inflicting chemical assault.” This not only leads to the demise of beneficial bacteria and the proliferation of harmful ones but, more critically, it systematically dismantles the gut mucus layer and epithelial tight junctions, which serve as physical and biochemical barriers. This results in increased intestinal permeability (“leaky gut”). Once the barrier is breached, bacterial endotoxins like lipopolysaccharide (LPS), normally confined to the gut lumen, can “translocate” into the bloodstream, causing “metabolic endotoxemia.” Circulating LPS is a potent signal that activates the systemic innate immune system. By binding to Toll-like receptor 4 (TLR4) on the surface of immune cells like macrophages, it activates the MyD88-dependent signaling pathway, ultimately leading to the nuclear translocation of NF-κB and the transcription and release of numerous pro-inflammatory cytokines (e.g., TNF-α, IL-1β, IL-6) (32). This state of persistent, low-grade systemic chronic inflammation is the final bridge that connects upstream dietary factors to the downstream pathology of nearly all NCDs. To counteract this diet-induced inflammatory and carcinogenic microenvironment, modern food science is urgently exploring advanced, targeted interventions. For instance, the integration of traditional “food and medicine homology” principles with modern scientific evidence is emerging as a crucial framework for modulating metabolic pathways and aiding in cancer prevention (33). Furthermore, effectively reversing UPF-induced dysbiosis requires precision medicine-food interventions; current research is increasingly leveraging multi-omics networks, AI-assisted profiling, and biomimetic or targeted microbial delivery systems to precisely repair the disrupted gut-microbiome axis (34, 35).

Collectively, current evidence indicates that higher consumption of ultra-processed foods is consistently associated not only with increased adiposity but also with elevated risks of several cancers (36)—including those of the head and neck, colorectum, and liver—as well as a greater incidence of depressive symptoms (37). These associations appear to extend beyond conventional nutrient-based explanations, implicating the degree and nature of industrial food processing as a key determinant of health outcomes. Mechanistically, the extensive structural and compositional modifications inherent in ultra-processing may disrupt ingestive behavior, metabolic homeostasis, gut microbial ecology, and neuroendocrine signaling through interconnected pathways. While convergent findings from epidemiological, clinical, and cohort studies from Monteiro strengthen this perspective (6, 36, 37), further longitudinal research in diverse real-world settings is warranted to clarify causal relationships and disentangle the independent contributions of food matrix disruption, additives, and nutrient profiles to chronic disease pathogenesis.

While the evidence presented outlines a compelling mechanistic framework linking food matrix integrity to metabolic and inflammatory pathways, it is important to acknowledge that much of this understanding derives from controlled feeding trials, animal models, and *in vitro* systems. The complex interplay between matrix structure, individual variability (e.g., genetics, baseline microbiota), and real-world dietary patterns remains incompletely characterized, underscoring the need for longitudinal human studies and standardized methodologies to isolate matrix effects from compositional confounders.

## 6 Discussion

### 6.1 The domino effect: a cascade from food matrix collapse to systemic disease

The chain of evidence integrated in this review clearly depicts a systemic “domino effect” triggered by the collapse of the food matrix. This pathophysiological cascade reveals that the harm of UPFs is far from an isolated nutritional issue; it is a powerful trigger capable of initiating a systemic disease process.

The first domino to fall is the structural collapse of the food matrix. This is the originator of all subsequent pathophysiological processes, the “hand” that pushes the entire chain.

It directly topples the second domino—behavioral and oral dysregulation (section 2). The industrially deconstructed soft matrix forces an accelerated eating rate and drastically reduces oro-sensory exposure, successfully bypassing the brain’s early satiety signals and the “cephalic phase response.”

Subsequently, the third domino—gastrointestinal endocrine dysregulation (section 3)—falls. Rapid gastric emptying and proximal small intestine absorption lead to the complete silencing of the “ileal brake” mechanism, with the secretion of key anorexigenic hormones GLP-1 and PYY being severely suppressed.

The failure of this endocrine feedback loop not only directly promotes excess energy intake but also creates the perfect conditions for toppling the fourth domino—core metabolic insult (section 4). A supraphysiological “nutrient flood” overwhelms the liver and peripheral tissues, driving severe hyperinsulinemia, *de novo* lipogenesis (DNL), and mitochondrial dysfunction, ultimately leading to the development of insulin resistance.

Following this, the fifth domino—gut ecological disaster (section 5)—succumbs. This is the inevitable result of the dual impact of “matrix deprivation” (lack of MACs) and “chemical assault” (food additives), manifesting as a famine for beneficial bacteria, disruption of the gut barrier, and the onset of “leaky gut.”

Finally, this domino inevitably pushes over the ultimate sixth domino—systemic chronic inflammation. Centered on metabolic endotoxemia (LPS leakage), this “fire” originating in the gut rapidly spreads throughout the body, converging all upstream local digestive and metabolic problems into a systemic, low-grade, chronic inflammatory state that provides the fertile ground for the pathology of almost all non-communicable diseases, from obesity and type 2 diabetes to cardiovascular disease.

This depiction of a “cascading reaction” leads to a powerful conclusion: the integrity of the food matrix is not merely one of many factors affecting a food’s “healthiness”; it is the cornerstone for maintaining holistic physiological homeostasis, from the behavioral to the cellular level. Once this cornerstone is undermined by industrial processing, the collapse of the entire health structure is only a matter of time.

Furthermore, it is crucial to acknowledge that UPFs represent a complex “causal bundle.” Matrix disruption rarely acts in isolation; it frequently co-occurs with hyper-palatability, high energy density, altered nutrient profiles, and the presence of additives. Rather than being the sole independent cause of NCDs, matrix collapse likely acts as a critical mediator and co-traveler that amplifies these other risk factors. Moreover, the landscape of food processing is highly heterogeneous. Some traditional processing methods (e.g., fermentation) alter food structure without clear detrimental health effects. Conversely, some minimally processed foods (e.g., liquid fruit smoothies) can be energy-dense and rapidly consumed despite lacking industrial formulation. Not all UPFs share the same degree of matrix destruction, highlighting the necessity for a more nuanced classification system that accounts for these exceptions and avoids binary oversimplifications.

### 6.2 Beyond traditional NCDs: the food matrix, gut-brain axis, and potential links to mental health

**FIGURE 2:**
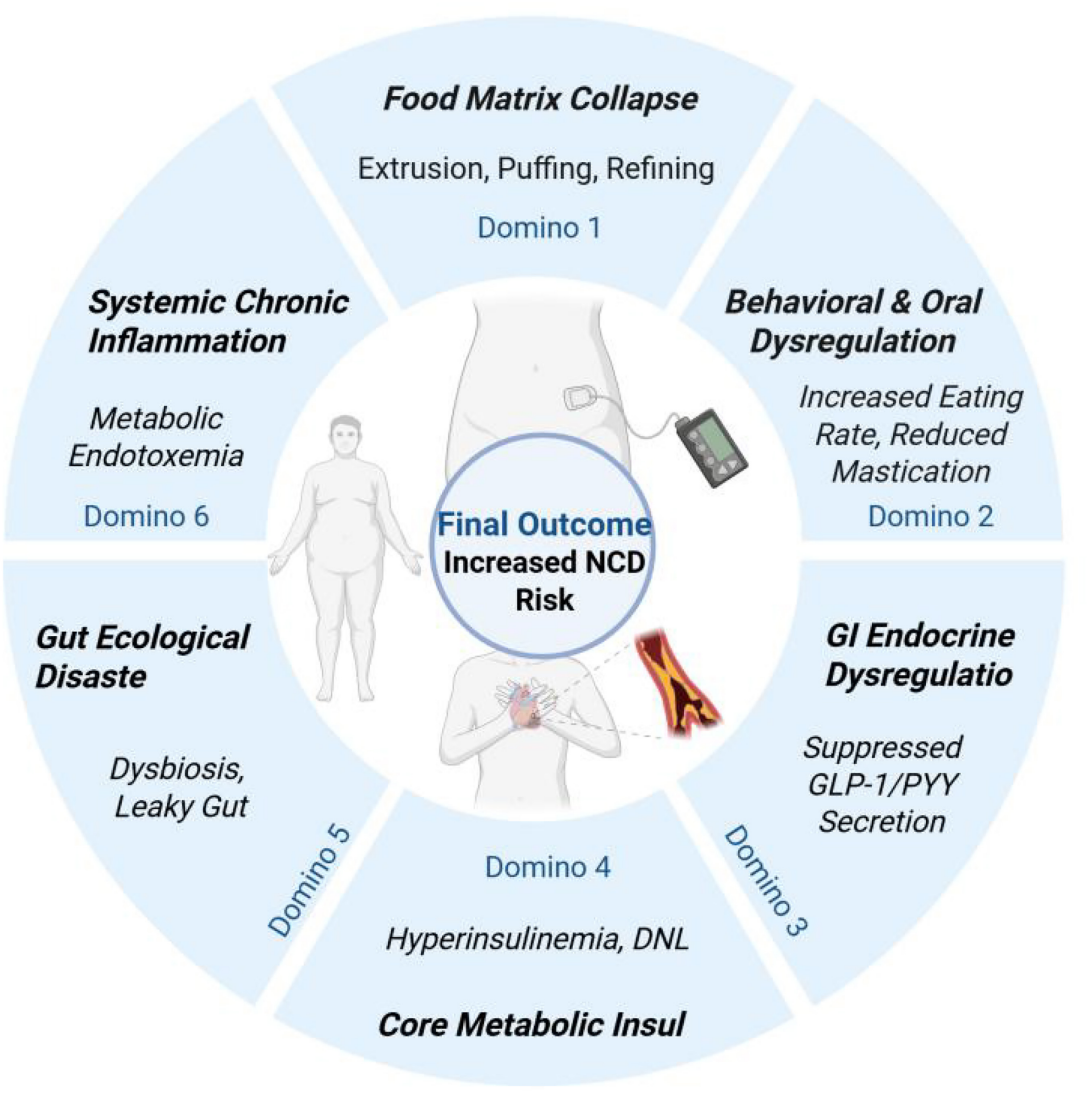
The proposed conceptual model of the domino effect cascade triggered by food matrix collapse.

The impact of food matrix collapse may extend far beyond traditional metabolic diseases. The gut dysbiosis and chronic inflammation described in this review are central to the emerging theory of the “gut-brain axis.” The gut microbiota communicates continuously and bidirectionally with the central nervous system through metabolic products (like SCFAs), neurotransmitters (like serotonin), and modulation of the vagus nerve (38). Dysbiosis and leaky gut driven by matrix collapse have been preliminarily linked to various neuropsychiatric disorders, including depression, anxiety, and even cognitive dysfunction (39). Therefore, a high-UPF dietary pattern may be pathogenic not only by impairing physical metabolism but also by disrupting the homeostasis of the gut-brain axis, becoming a risk factor for poor mental health. This perspective significantly broadens the potential application of the food matrix concept, suggesting that future research should pay closer attention to the long-term effects of dietary processing on neurological and mental health, see as Figure 2.

### 6.3 A critique of “healthy” ultra-processed foods: the limits of fortification and the fallacy of “nutritionism”

In response to growing public health concerns about UPFs, the food industry has adopted a typical strategy: launching a plethora of new-generation UPFs that are “nutritionally optimized” or “functionally fortified.” These products, such as high-protein energy bars, fiber-enriched cookies, and meal replacement shakes fortified with vitamins and minerals, base their marketing claims on a reductionist ideology known as “nutritionism”—the belief that the value of a food is equivalent to the sum of its constituent nutrients. However, viewed through the “food matrix” framework constructed in this review, the health claims of these products are fundamentally flawed.

These so-called “healthy” UPFs, even with seemingly impeccable nutrition labels, share a fundamental defect that cannot be remedied by “nutritional addition”: their physical matrix remains thoroughly collapsed. Take high-protein bars, for instance. Their protein source is typically highly purified whey or soy protein isolate. The absorption kinetics of these “fast proteins” are vastly different from the “slow proteins” found in whole foods (like a piece of steak or an egg), which are encapsulated by a matrix of collagen and other components. They are rapidly digested and absorbed, potentially causing a transient spike in blood ammonia levels, increasing the burden on the kidneys, and providing a much shorter duration of satiety than their whole-food counterparts.

Similarly, UPFs advertised as “rich in fiber” often contain single, refined soluble fibers extracted from natural plants, such as inulin, fructo-oligosaccharides, or polydextrose. This practice fails to replicate the complex, three-dimensional physical barrier found in natural whole grains or legumes, which is composed of both insoluble fibers (like cellulose and lignin) and soluble fibers (like pectin). Such refined fibers are largely ineffective at slowing the rapid absorption of starches and sugars, nor can they provide the structurally diverse fermentation substrates (MACs) that a wide variety of gut microbes depend on for survival.

The most telling example is the meal replacement shake. Often marketed as “nutritionally balanced” weight-loss solutions, these products are the ultimate embodiment of the food matrix collapse philosophy. Their fully liquefied, pre-digested form allows all nutrients—carbohydrates, proteins, and fats—to pass through the stomach at maximum speed and be absorbed “in a flash” in the upper small intestine. This design maximally bypasses all endogenous satiety regulation mechanisms (from oral chewing to the ileal brake) and inflicts the most severe metabolic shock on the body.

Therefore, a critical reflection on “healthy” UPFs is the final logical extension of this review’s core argument: the health value of food arises from the organic unity of its structure and composition. Merely playing an “arithmetic game” with nutrients on top of an already collapsed industrial matrix is a misleading marketing strategy, not a genuine product innovation aimed at promoting long-term health. This profoundly reveals the critical importance of moving beyond the fallacy of “nutritionism” and returning to a respect for the natural integrity of food.

## 7 Conclusion and future perspectives

### 7.1 Conclusion

In summary, by systematically integrating multi-dimensional evidence, this review reaches a clear and significant conclusion: the fundamental threat of ultra-processed foods to global health is driven by far more than their well-known adverse nutrient profiles. More profoundly, it originates from the industrial collapse of their physical “food matrix.” This structural disintegration is not a trivial physical change but the “first domino” that initiates a top-down, systemic cascade of dysregulation.

This pathophysiological process begins in the mouth, where the collapsed matrix bypasses the first line of behavioral and neurophysiological satiety defense by accelerating eating rates and diminishing sensory exposure. Subsequently, in the gastrointestinal tract, rapid digestion and absorption lead to the silencing of the “ileal brake” mechanism, suppressing the secretion of key anorexigenic hormones. This endocrine dysregulation opens the floodgates for subsequent metabolic turmoil: a supraphysiological “nutrient flood” impacts the liver and peripheral tissues, driving insulin resistance and *de novo* lipogenesis. Finally, this storm creates an “ecological disaster” within the gut, where an impoverished matrix and chemical additives destroy the gut microbiota and physical barrier, igniting systemic, low-grade chronic inflammation centered on “metabolic endotoxemia”—the common pathophysiological soil for nearly all non-communicable diseases.

It should be noted, however, that while the proposed cascade is supported by converging lines of experimental and observational evidence, direct causal validation in diverse human populations—particularly under free-living conditions—is still limited. Many mechanistic insights rely on short-term controlled trials or surrogate endpoints, and the relative contribution of matrix disruption versus specific additives or nutrient profiles remains challenging to disentangle in real-world dietary contexts. Therefore, this review strongly calls for a profound paradigm shift in nutritional science, public health policy, and clinical practice. We must move beyond the limitations of “nutritionism” and shift our focus from an isolated perspective of “what is in our food” to a holistic view of “what has been done to our food.” Only by placing the degree of food processing, particularly the integrity of its physical matrix, at a core position of equal importance to nutrient composition can we truly understand and effectively combat the global health crisis driven by ultra-processed foods.

### 7.2 Future research directions

To transform the “food matrix” concept from a potent theoretical framework into a scientific tool that can effectively guide public health practice and personalized nutrition, future research should be systematically pursued in the following four key directions:

(1) Methodological innovation and standardization: to rigorously test the proposed “Domino Effect” cascade, future clinical trials should employ sequential, multi-system measurement designs. Researchers should feed participants isocaloric, nutrient-matched meals that differ strictly in matrix integrity (e.g., intact whole grains vs. heavily extruded equivalents). A robust study would simultaneously quantify chewing kinematics and eating rate (testing Domino 2), measure postprandial kinetics of GLP-1 and PYY via frequent blood sampling (testing Domino 3), assess continuous glucose profiles and lipogenesis markers (testing Domino 4), and monitor acute changes in systemic inflammatory cytokines (testing Domino 6). Such integrated designs are essential to isolate the matrix effect from compositional confounders.

(2) Multi-omics deep exploration and mechanistic validation: future studies should move beyond simple 16S rRNA sequencing to higher-resolution metagenomics, metatranscriptomics, and metaproteomics to precisely delineate the true impact of different food matrices on the function (not just composition) of the gut microbiome. Combined with host metabolomics and proteomics, we can construct a comprehensive “host-microbe co-metabolic network” map. This will allow for a dynamic and complete picture of the changes in endogenous biological signals (such as SCFAs, secondary bile acids, LPS) following the ingestion of different food matrices, ultimately leading to the discovery of novel biomarkers for early warning and intervention.

(3) Special populations and clinical translation research: the effects of the food matrix are likely not uniform but exhibit significant individual and group differences. Therefore, future research must focus on the impact of matrix effects on key life-stage populations (e.g., infants, pregnant women, the elderly) and individuals with specific disease states (e.g., patients with type 2 diabetes, NAFLD, or IBD). Clinical trials are especially needed to explore whether dietary interventions centered on “preserving the food matrix” (e.g., replacing refined grains with whole grains, whole fruits for fruit juice) can serve as an effective adjuvant therapy to improve clinical outcomes and quality of life in these high-risk groups.

(4) Food science and “gentle processing” technology innovation: the ultimate goal of this review is not to negate all food processing but to guide it in a healthier direction. Therefore, the field of food science should invest heavily in the research and evaluation of “minimal processing” or “structure-friendly” technologies. These technologies, such as high hydrostatic pressure, pulsed electric fields, freeze-drying, and novel extrusion techniques, aim to preserve the natural cellular structure and matrix integrity of food to the greatest extent possible while ensuring food safety and convenience. Evaluating the long-term satiety and metabolic health effects of foods produced with these innovative technologies will be the key bridge connecting basic nutritional science with the future of the healthy food industry.
